# Impact of millimeter‐scale target motion on the precision of spatially fractionated radiation therapy using proton minibeams

**DOI:** 10.1002/mp.70638

**Published:** 2026-08-18

**Authors:** Oxana Actis, Cristian Fernandez‐Palomo, Verdiana Trappetti, Michele Togno, Paolo Pellicioli, Dominic Leiser, David Meer, Valentin Djonov

**Affiliations:** ^1^ Center for Proton Therapy Paul Scherrer Institute Villigen PSI Aargau Switzerland; ^2^ Institute of Anatomy University of Bern Bern Switzerland

**Keywords:** beam delivery, minibeams, proton therapy

## Abstract

**Background:**

While pencil beam scanning (PBS) with motion mitigation such as re‐scanning, breath hold, or gating is well established for treating moving targets in clinical practice, the precision for Spatially Fractionated Radiation Therapy (SFRT) using proton minibeams introduces presents a unique challenge. The sub‐millimeter precision required for SFRT cannot be achieved with typical PBS spot sizes (millimeters), necessitating multi‐slit beam collimation, which reduces the dose rate and reintroduces motion as a critical issue. In this study, we report on our experience with minibeam irradiations of chorioallantoic membranes (CAM) of chick embryos and discuss potential solutions to the motion problem.

**Methods:**

The first experiment was conducted at a PBS gantry using 130 MeV protons and a 6 cm thick copper multi‐slit collimator with 0.3 mm wide slits and 1 mm spacing. To achieve a target peak dose of 30 Gy, CAM samples were irradiated with more than 10 re‐scans. Due to a 91% beam loss in the collimator, the total irradiation time extended to two min.

**Results:**

Post‐irradiation analysis using gafchromic film revealed smearing of the minibeam profiles, while biological tissue analysis uncovered unexpected and disrupted dose effects, likely caused by irregular CAM movement during irradiation. A subsequent experiment, incorporating additional setup optimizations such as reduced air gap and increased dose rate, shortened the irradiation time to below one min. This led to sharper dose profiles with negligible variation in valley dose and peak width, although a reduction in peak dose of 8%–13% was still observed. This variation is considered acceptable for our biological experiments.

**Conclusions:**

For clinical applications, minibeam re‐scanning is likely to be feasible only under ultra‐high dose rate conditions (e.g., those required to induce the FLASH effect), where sufficiently short irradiation times minimize motion‐induced dose degradation.

## 1 INTRODUCTION

Proton therapy using pencil beam scanning (PBS) has become a standard modality in clinical practice due to its ability to deliver conformal dose distributions and exploit the Bragg peak for healthy tissue sparing. For the treatment of moving targets, motion mitigation techniques such as rescanning, breath‐hold and gating are well established in conventional PBS in such conditions.

Proton minibeam therapy (pMBT) introduces an additional spatial fractionation layer by dividing the beam into sub‐millimeter‐sized minibeams. Preclinical evidence suggests that pMBT can enhance normal tissue tolerance and increase the therapeutic window.^1^, ^2^, ^3^, ^4^, ^5^ However, conventional PBS spot sizes (millimeters) are too large to directly generate minibeams, requiring collimation that significantly reduces dose throughput (collimated peak dose with respect to uncollimated homogeneous flat field dose) or additional beam focusing.^6^, ^7^, ^8^, ^9^ Combining pMBT with moving targets introduces new challenges due to interplay effects and limited dose rate capabilities, which can lead to dose blurring and reduced peak‐to‐valley dose ratios (PVDR).

In this study we demonstrate the potential problems of collimated proton minibeam delivery to moving targets using a chick embryo CAM model.^10^ We analyze beam delivery parameters, motion effects, and potential mitigation strategies for future pMBT applications.

## 2 MATERIALS AND METHODS

### 2.1 Beam delivery system and biological model

**FIGURE 1:**
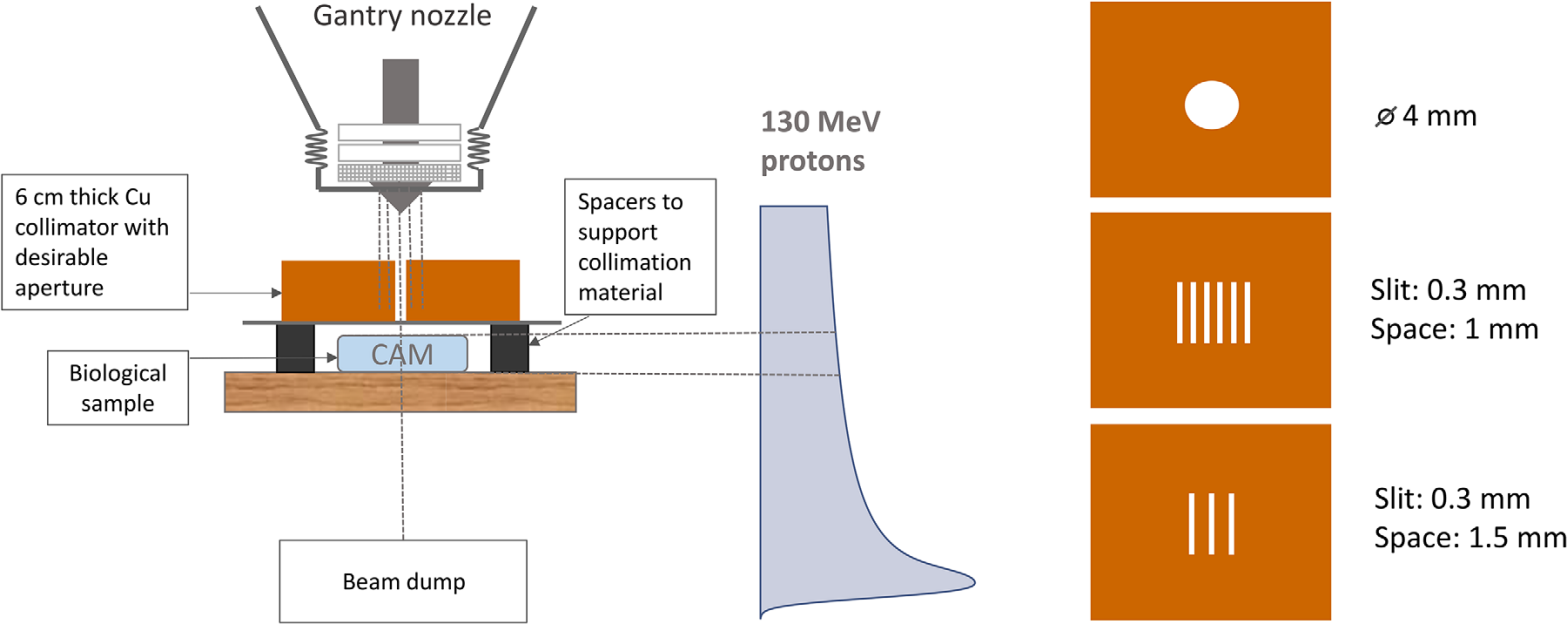
Experimental setup and collimator configurations.

We performed our experiments at the PBS gantry designed for fast 3D scanning, delivering energies between 70 and 230 MeV and beam sigma ranging from 6.5 to 2.5 mm. The system delivers proton beams of 10^5^ to 10^8^ protons per spot with lateral scanning speeds of 2 cm/ms and 0.5 cm/ms in the two transverse directions of 12 cm x 20 cm. Due to the absence of direct minibeam focusing we used a 6 cm thick copper collimator with various apertures: round aperture of 4 mm diameter, 6‐slits of 10 mm x 0.3 mm and 1.3 mm c‐t‐c distance and 3‐slit collimator with the same slit‐size, but 1.8 mm c‐t‐c distance. Figure 1 illustrates the setup and collimator apertures.

We used 130 MeV proton beam irradiating the target in the plateau region. Considering that 130 MeV beam sigma at our system is around 3.5 mm we had to apply a scanned field to achieve a homogeneous dose distribution with sharp beam penumbra after collimation. The maximum peak dose we delivered to the target varied between 30 and 50 Gy which we could not deliver in a single scan due to limitations in our machine configuration. Thus, we had to repeat the scan several times, basically utilizing the layer re‐scanning technique.^11^

Our biological model was the chick embryo chorioallantoic membrane (CAM) chosen for its high accessibility and sensitivity to radiation‐induced biological effects. It exhibits physiological movement due to rhythmic embryonic muscle contractions and the pulsatile action of the heartbeat driving blood flow through its extensive vascular network. Samples were placed downstream from the collimator at distances of 39 mm (Experiment 1) and at a minimal experimentally possible distance of 17 mm (Experiment 2).

### 2.2 Dosimetry and Monte Carlo simulations

Our therapy system delivers the prescribed dose with an online monitoring accuracy of better than 1%.^12^ For setups involving collimators, however, we validated the actual dose delivery at the target. First, we cross‐calibrated an in‐house developed scintillation‐based detector against a microDiamond 60019 ion chamber (PTW, Freiburg, Germany) by applying a homogeneous, mono‐energetic dose field without a collimator. This detector consists of a Gd_2_O_2_S screen and is imaged with a CMOS sensor. We measured the integral dose of the radiation field for 1, 5, 10, and 15 Gy, confirming a linear relationship between dose output and the number of protons delivered at the selected energy. For doses exceeding 15 Gy, the number of protons per spot was scaled accordingly. Once the detector was calibrated, we installed collimators at the air gap defined by our experimental setup and measured dose throughput for all experimental apertures.

To further optimize the experimental configuration and assess uncertainties, we performed Monte Carlo simulations using TOPAS (Tool for Particle Simulation).^13^ The primary parameters of interest were the PVDR and the dose gradient. By simulating a scanned proton beam grid, we determined that a collimator thickness of 6 cm represents a good compromise between penumbra sharpness and acceptable proton dose throughput. Using TOPAS, we also evaluated the effects of collimator tilt and air gap. Vertical collimator tilt was maintained below 0.1°, resulting in a negligible impact on the final dose, whereas TOPAS simulations revealed that the air gap strongly affected the peak dose. This finding established a key optimization parameter for our second measurement campaign.

### 2.3 Experimental procedure

We conducted two experimental campaigns:

**Experiment #1**: We aligned the CAM sample at the iso‐center using a collimator‐to‐target air gap of 39 mm. For all collimators re‐scanning was used to reach cumulative peak doses of 30 and 50 Gy. For multi‐slit aperture the required irradiation time was above 2 min per field.

**Experiment #2**: We reduced the scanning field size for the multi‐slit collimator (3 slits instead of 6) and used a shorter air gap of 17 mm (minimum possible with our setup). We also increased the beam delivery intensity by 50% to reduce the total dose delivery time to under 1 min.

In addition, we repeated the CAM irradiations using experimental setup 2 placing the gafchromic films on top of the CAM with the objective to analyze not the biological tissue but the films. Afterwards, we compared these measurements to images we acquired in static conditions using the same setup parameters. To quantify the CAM motion, we recorded videos for three embryos of various sizes, with recording durations matching the experimental irradiation time. Several markers were placed on the CAM surface, and we analyzed their displacement (details in supplementary materials S‐1).

## 3 RESULTS

With the 4 mm circular collimator we measured the dose throughput of 100%, and at our standard dose rate of 1 Gy/s. In contrast, throughput using the 0.3 mm multi‐slit collimator (Experiment 1) was only 9%, resulting in irradiation time of > two min for 30 Gy peak dose in the target. By reducing the air gap from 39 to 17 mm, increasing the dose rate from 1 Gy/s to 1.5 Gy/s and decreasing the irradiation area by reducing the number of slits in the optimized collimator, the same dose could be delivered in less than one min.

**FIGURE 2:**
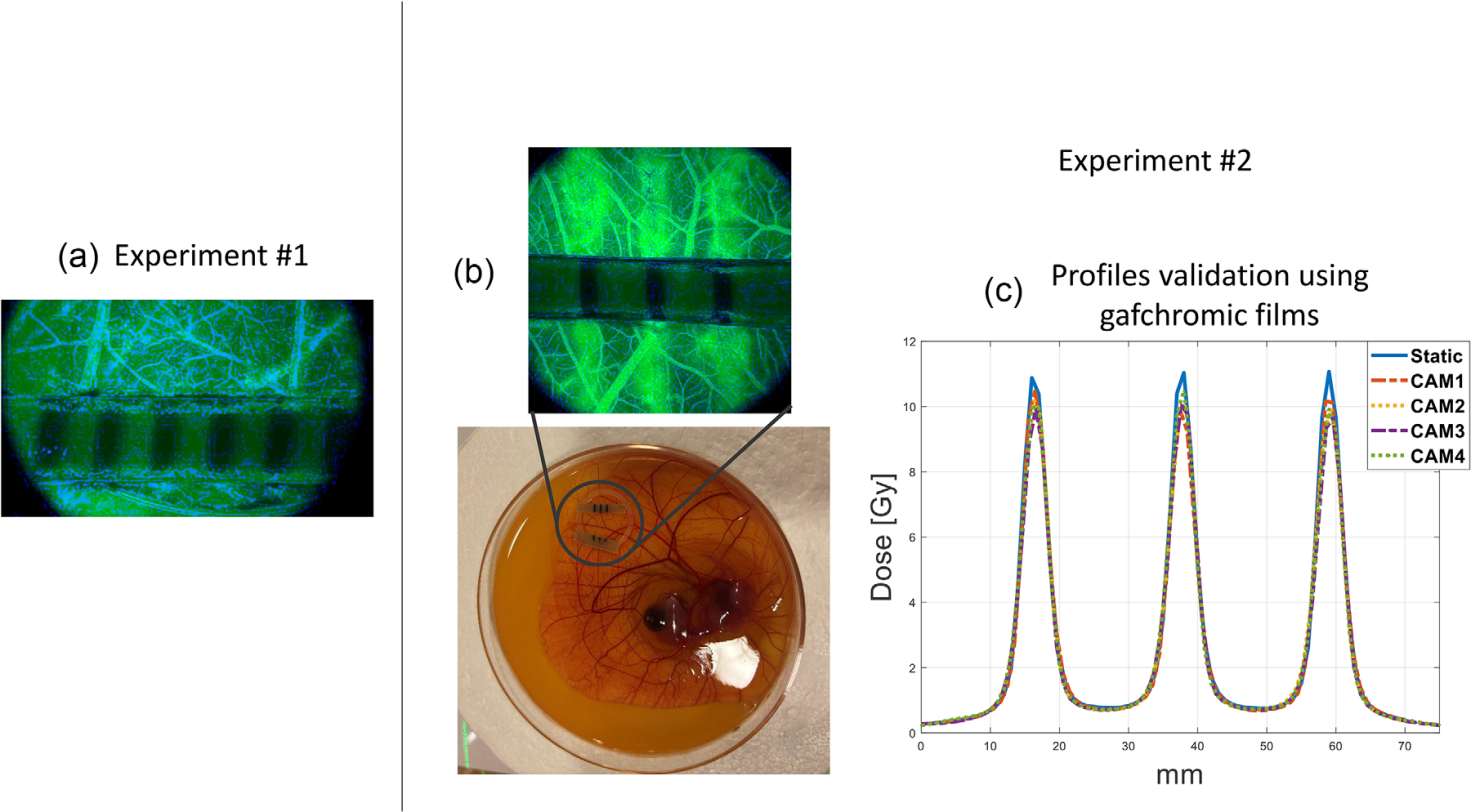
(a) Gafchromic films placed on top of the CAM (Experiment 1); (b) gafchromic film from Experiment 2; (c) profiles measured with gafchromic films for static irradiation and films irradiated on the CAM surface.

Experiment 1 showed that a 2 min irradiation of the CAM produced clear proton minibeams, but with a visible lateral smearing of the peak doses (Figure 2a). In Experiment 2, we optimized the setup which led to a visible improvement in the dose distribution and sharper minibeams (Figure 2b). Reducing the air gap increased the PVDR by 45%, as measured using a scintillation‐based detector. The optimized setup produced almost no variation in valley dose or peak width but resulted in a peak dose reduction of 8%–13%, which we consider acceptable for our biological experiments.

By recording the CAM movement for over a min, we were able to track how markers on the CAM moved over time. We observed the average motion displacement of 40 µm and a maximum displacement of 120 µm, which we believe led to peak dose distortion in Figure 2c. Although the motion amplitudes were small compared with typical clinical scenarios, the sub‐millimeter scale of the minibeam structure was sensitive to such displacements.

## 4 DISCUSSION

This study demonstrates that pMBT offers promising potential for sparing normal tissue and can achieve effective dose distributions in the CAM model. However, its performance is highly sensitive to target motion and beam delivery parameters. Our setup optimization, including a reduced air gap (leading to improved penumbra) and an increased dose rate, significantly enhanced beam sharpness and reduced delivery time, with remaining peak dose reductions (8%–13%) considered acceptable for the experimental objectives.

The primary challenge of this study was the presence of CAM motion, which introduced interplay effects that may have adversely impacted dose distribution. We characterized two main motion sources: blood‐vessel pulsation (mean displacement: 40 µm) and intrinsic embryo movement (maximum amplitude: 120 µm, duration: ∼2 s). Although we minimized vascular motion by positioning the film on the capillary plexus, the unpredictable nature of embryo movement may lead to minibeam smearing, potentially resulting in a brief increase in penumbral dose and a reduction in peak dose. While no radiation toxicity was observed, the biological implications of these motion‐induced dose variations remain to be elucidated and warrant further investigation. More studies like^14^ have to be carries on.

A key limitation arises from beam losses due to collimation, which results in prolonged delivery times that are not compatible with the irradiation of moving targets. Several strategies may help to overcome these challenges. Ultra‐high dose rates, such as those used in FLASH radiotherapy, could shorten delivery times sufficiently reducing the interplay effect. Improved experimental setup designs that increase transmission efficiency would also be beneficial, enabling higher peak‐to‐valley ratios and faster dose delivery. Finally, the development of additional sub‐millimeter beam focusing would be beneficial for minimizing beam loss and the possibility of combinations with standard PBS motion management approaches.

## 5 CONCLUSIONS

Our initial proton minibeam experiments on moving CAM model samples that current PBS and collimation‐based delivery approaches are not robust against target motion. While setup optimizations can partially mitigate motion effects, fundamental limitations in dose rate and beam shaping technology must be overcome. Future developments in high‐efficiency collimators, FLASH‐capable PBS systems, and suitable motion compensation are essential to translate pMBT to clinical scenarios involving moving organs.

## CONFLICT OF INTEREST STATEMENT

The authors declare no conflicts of interest.

## Supporting information



Supporting Information
