# Supplementary material for: Impact of millimeter‐scale target motion on the precision of spatially fractionated radiation therapy using proton minibeams
Source: Med Phys. 2026 Aug 18;53(9):e70638. doi: 10.1002/mp.70638 (PMC13486916; doi:10.1002/mp.70638)
Supplement: Supplementary file 1 [file MP-53-0-s001.doc]

# **S-1: Supplementary Material: Methods**

**Figure S-1.** The ring with diameter of 1.5 cm and Marker 2 (on the left) is placed on top of the CAM to indicate the typical area selected for irradiation in our experiment. Two other markers are placed on purpose in the yock area closer to embryo. The right plot shows the displacement for all three markers over time.

**Methods:**

To quantify the overall motion, we recorded a 1-minute video (corresponding to the typical irradiation time) for three embryos. Markers were placed on the CAM surface, and their displacement was analyzed after registration. While the observed motion was irregular and non-periodic, the displacement amplitudes in the targeted area (marked with a ring in Figure S-1, left) consistently fell within the range of 2–120 µm, with a maximum amplitude of 120 µm and an average amplitude of 40 µm. For markers placed closer to the embryo and larger blood vessels, displacement amplitudes were significantly higher. These findings suggest that, although motion is present in our region of interest, its magnitude remains within a quantifiable and manageable range for our experimental purposes.
